# Distorted Beliefs about Luck and Skill and Their Relation to Gambling Problems and Gambling Behavior in Dutch Gamblers

**DOI:** 10.3389/fpsyg.2017.02245

**Published:** 2017-12-22

**Authors:** Megan E. Cowie, Sherry H. Stewart, Joshua Salmon, Pam Collins, Mohammed Al-Hamdani, Marilisa Boffo, Elske Salemink, David de Jong, Ruby Smits, Reinout W. Wiers

**Affiliations:** ^1^Mood, Anxiety, and Addiction Comorbidity Laboratory, Department of Psychology and Neuroscience, Dalhousie University, Halifax, NS, Canada; ^2^Department of Psychiatry, Dalhousie University, Halifax, NS, Canada; ^3^Department of Community Health and Epidemiology, Dalhousie University, Halifax, NS, Canada; ^4^Addiction, Development, and Psychopathology Lab, Department of Psychology, University of Amsterdam, Amsterdam, Netherlands

**Keywords:** cognitive distortions, gambling behavior, gambling problems, luck, skill, measurement

## Abstract

Gamblers’ cognitive distortions are thought to be an important mechanism involved in the development and maintenance of problem gambling. The Gambling Cognitions Inventory (GCI) evaluates two categories of distortions: beliefs that one is lucky (i.e., “Luck/Chance”) and beliefs that one has special gambling-related skills (i.e., “Skill/Attitude”). Prior psychometric evaluations of the GCI demonstrated the utility of both subscales as measures of distortions and their concurrent relations to gambling *problems* among Canadian gamblers. However, these associations have not yet been studied in gamblers from other cultures nor have relationships between the GCI and indices of gambling *behavior* been investigated. In addition, the predictive validity of the GCI scales have not been evaluated in studies to date. The present study investigated the validity of the GCI as a measure of cognitive distortions in a sample of 49 Dutch gamblers by examining its concurrent and prospective relationships to both gambling *problems* (as measured through a standardized nine-item questionnaire assessing gambling-related problems) and *behaviors* (as measured through two variables: days spent gambling and time spent gambling in minutes) at baseline and over 1-month and 6-month intervals. The GCI subscales were internally consistent at all timepoints, and moderately to strongly inter-correlated at all timepoints. Each subscale correlated with an independent dimension of gambling both concurrently and prospectively: Luck/Chance was related to greater gambling *problems* and Skill/Attitude was related to greater gambling *behavior*. Thus, the two GCI subscales, while inter-correlated, appear to be related to different gambling outcomes, at least among Dutch gamblers. Moreover, the first evidence of the predictive validity of the GCI scales was demonstrated over a 1-month and 6-month interval. It is recommended that both types of cognitive distortions be considered in research and clinical practice to fully understand and address individual risk for excessive and problematic gambling.

## Introduction

Problem gambling is an important public health concern in many countries. In North America, 3.8% of individuals will experience symptoms of problem gambling within their lifetime, while 1.8% will exhibit more severe symptoms of past year gambling disorder (Jacques et al., 2000). Specifically in Canada and the United States, past year prevalence rates of problem gambling averaged 1.8 and 3.2%, respectively (Williams et al., 2012). Those in European countries experience similar levels of gambling problems to those in North America, with 2.0–3.5% exhibiting symptoms of problem gambling and 1.2–3.2% displaying more severe symptoms of a gambling disorder (Becona, 1996). Past year gambling rates differ between European countries, however, ranging from 0.5% in Netherlands and Denmark to 2.8% in Belgium (Williams et al., 2012). Gambling often results in financial losses for gamblers. In Canada, for example, the industry acquires a yearly average of approximately $237 per capita compared to $372 per capita in the United States and €51.76 per capita in Netherlands (Global Betting and Gaming Consultants, 2002). In addition to financial losses, disordered gambling creates adverse familial, societal, and psychological consequences. Such consequences underscore the importance of further investigation into its underlying mechanisms. Though many hypothesized pathways to a gambling problem exist, cognitive distortions are thought to be an important mechanism involved in the development and maintenance of problem and disordered gambling (Ladouceur and Walker, 1996).

### Cognitive Distortions

Gambling-related cognitive distortions are central to the cognitive theory of gambling disorder. The cognitive theory of gambling disorder posits that cognitive distortions, also referred to as erroneous beliefs or fallacies, are involved in the development of, and serve to maintain, problem gambling (Ladouceur and Walker, 1996; Ladouceur, 2004). Broadly, these cognitive distortions are a set of false or exaggerated underlying beliefs that influence the automatic thoughts and behaviors a gambler experiences or displays during a gambling session (Ladouceur and Walker, 1996). These beliefs are thought to arise from the gambler’s misperception of randomness, prompting the individual to believe he or she can exert control over and correctly predict the outcome of a chance-determined game. Motivated by the opportunity for monetary gain, the gambler’s cognitive distortions prompt strategizing around the development of his or her gambling-related skill in an attempt to increase the likelihood of winning. The cognitive theory of problem gambling emphasizes that these faulty beliefs perpetuate gambling disorder by impacting the gambler’s understanding of randomness, perceived control over the game, attributions of skill, motives for continued engagement in gambling, and perceived reasons for gambling losses (Breen et al., 2001).

### The Gambling Cognitions Inventory

In a Canadian study, Holub (2003) developed a 40-item measure of gambling-related cognitive distortions called the Gambling Cognitions Inventory (GCI). McInnes et al. (2014) validated the GCI as a measure of gambling-related cognitive distortions in four different samples of Canadian problem and disordered gamblers. Confirmatory factor analysis confirmed a two-factor structure–beliefs one has special gambling-related skills (i.e., Skill/Attitude factor) and beliefs one is lucky (i.e., Luck/Chance factor). Each factor exhibited good internal reliability, with alphas ranging from 0.86 to 0.92 for the Skill/Attitude subscale and 0.83 to 0.90 for the Luck/Chance subscale. Moreover, the GCI showed good convergent validity with other measures of gambling distortions and with measures of gambling problems.

noted the two factors of the GCI closely mimic two of the most commonly studied fallacies in the gambling literature, the Illusion of Control (i.e., belief that one can control the outcome of a chance-determined game; Langer, 1975) for Skill/Attitude and the Gambler’s Fallacy (i.e., belief that frequent losses will be followed by an imminent win; Toneatto, 1999) for Luck/Chance. Moreover, McInnes et al. (2014) note the GCI is unlike other measures of distortions in one important respect. As opposed to other measures of gambling-related cognitive distortions, which often assess both gambling-related distortions and other types of gambling-related cognitions, the GCI specifically assesses gambling-related cognitive distortions. For example, along with assessing gambling-related cognitive distortions, the Gambling Related Cognitions Scale also measures gambling expectancies (e.g., “gambling makes things seem better”) and the gambler’s perceived capacity to stop gambling (e.g., “I can’t function without gambling”; Raylu and Oei, 2004a, p. 768).

### The Importance of Examining Gambling *Problems* and Gambling *Behaviors*

In their study, McInnes et al. (2014) found both types of cognitive distortions (i.e., Luck/Chance and Skill/Attitude), as indexed by the GCI scales, were related to severity of gambling *problems*. However, akin to other addictive behaviors, the importance of examining both *problems* and *behaviors* as distinct outcomes has been well established. Sadava (1985) found alcohol consumption behavior and alcohol-related problems were only moderately correlated constructs (*r*’s = 0.08–0.50), suggesting behaviors and problems are overlapping yet unique alcohol outcome dimensions. Moreover, certain variables independently relate to alcohol use *behaviors* and alcohol-related *problems*. For example, Stewart et al. (2006) found social anxiety (i.e., an intense fear of embarrassment or negative evaluation in social situations resulting in an avoidance of such situations; American Psychiatric Association, 2013) was positively related to drinking *problems*, yet negatively related to drinking *behavior*. In other words, socially anxious individuals seldom drink but when they do drink, they are likely to experience alcohol-related problems (Stewart et al., 2006). This finding demonstrates that drinking problems and drinking behavior are distinct and should be studied as such.

Like alcohol, disordered gambling is best understood by examining the underlying gambling behaviors that engender gambling problems (Walker et al., 2006). Examining gambling behaviors is relevant to understanding disordered gambling—targeting gambling behaviors in treatment often resolves gambling problems. Akin to the alcohol literature, though inextricably linked, gambling behaviors (e.g., gambling frequency, time spent gambling, money spent gambling) and gambling-related problems (i.e., negative outcomes from gambling such as financial and relationship problems) are moderately, but not highly, inter-correlated (*r*’s = 0.34–0.61; Joukhador et al., 2004; Fischer and Smith, 2008; Flack and Morris, 2015). Although these two dimensions of gambling show some overlap, they are distinct, thus meriting an independent investigation of each dimension to fully comprehend the nature of disordered gambling. For example, Fischer and Smith (2008) examined the differential relationship of several impulsivity-related constructs to gambling *behavior* and gambling *problems*. The impulsivity constructs examined included “urgency” (i.e., the propensity to behave impulsively when upset) and “sensation seeking” (i.e., the propensity to pursue experiences that are novel or stimulating; Whiteside and Lynam, 2001). Although gambling *behavior* (as measured by gambling frequency) and gambling *problems* [as measured by the South Oakes Gambling Screen (SOGS); Lesieur and Blume, 1987] were moderately inter-correlated (*r* = 0.34), urgency was positively correlated solely with gambling *problems* while sensation seeking was positively correlated solely with gambling *behavior*. Evidently, examining either gambling problems *or* gambling behaviors alone does not paint a complete picture of disordered gambling. Given these findings, it is important to include measures of gambling *behavior* in addition to measures of gambling *problems* as outcome variables when examining the validity of any measure of gambling-related cognitive distortions.

#### Cognitive Distortions and Gambling *Problems*

Gambling-related cognitive distortions have been consistently found to be related to gambling *problems*. Previous literature has found a positive relationship between gambling-related cognitive distortions and problem gambling severity, where those with a greater severity of gambling problems endorse significantly more cognitive distortions compared to those with less severe gambling problems (Cunningham et al., 2014). For example, disordered gamblers and problem gamblers endorse considerably more cognitive distortions compared to both non-problem and social gamblers (Toneatto, 1999; Joukhador et al., 2004; Myrseth et al., 2010), even after controlling for genetic and environmental factors (Xian et al., 2009). While it is evident that gambling-related cognitive distortions and gambling problems are related, far less research examines the relation of gambling-related cognitive distortions to gambling behaviors.

#### Cognitive Distortions and Gambling *Behaviors*

A few studies have established a relationship between gambling-related cognitive distortions and gambling behaviors. In a sample of machine gamblers, for example, Joukhador et al. (2004) found those with greater superstitious beliefs around gambling spent more time gambling and engaged in more gambling sessions each week compared to those with less superstitious beliefs. Using data from five gambling prevalence studies, Miller and Currie (2008) examined the relationship between gambling-related cognitive distortions and risky gambling behaviors (i.e., borrowing money to gamble, returning to gamble to recoup prior losses, and betting more money than one can afford) in 11,652 Canadian gamblers. They found that gambling-related cognitive distortions and risky gambling behaviors were positively related. Specifically, those who endorsed a higher degree of gambling-related cognitive distortions engaged in significantly more risky gambling behaviors than those who endorsed a lesser degree of cognitive distortions. Yakovenko et al. (2016) sought to assess the temporal directionality of the relationship between cognitive distortions and gambling behaviors (i.e., gambling frequency, money spent, number of games played). They recruited 1,372 participants with varying degrees of gambling severity (i.e., 1,288 non-gamblers, 43 low-risk gamblers, 41 disordered gamblers) and found distortions, as indexed by the Gambling Fallacies Scale (Williams, 2003, Unpublished), predicted increases in gambling behaviors over time. In sum, to understand how cognitive distortions contribute to the development and maintenance of disordered gambling, we must elucidate their relationship to both gambling problems and gambling behaviors.

### Cultural Differences in Gambling Distortions

While these studies further our understanding of the role of cognitive distortions in gambling-related problems and behaviors, this body of empirical research has been predominantly performed with North American samples (Raylu and Oei, 2004b). Varying values and beliefs across cultures may be reflected in varying gambling-related cognitive distortions across cultures, in turn contributing to cultural differences in gambling behaviors and levels of gambling problems (Raylu and Oei, 2004b).

Gambling is defined differently across cultures (Dickins and Thomas, 2016). In their review of the literature, Dickins and Thomas (2016) note that the definition of gambling is molded by the collective attitude, acquired through cultural customs. These alternative definitions of gambling result in certain gambling practices being viewed as acceptable in some cultures yet unacceptable in others. Accompanying these cultural beliefs are various risk and protective factors that help prompt or protect against disordered gambling, with these influences potentially varying across cultures (Oei and Goh, 2015). For example, self-perceived resilience has been linked to greater gambling problems among Chinese gamblers who endorse greater gambling-related cognitive distortions (Oei and Goh, 2015) yet less severe gambling problems in Canadian gamblers (Lussier et al., 2007). It has been speculated that cultural beliefs which favor gambling, such as those based on superstition, fate, luck, and chance, might contribute to cross-cultural differences in disordered gambling by means of encouraging and normalizing gambling involvement (Raylu and Oei, 2004b; Papineau, 2005; Oei and Goh, 2015). While beliefs in luck and chance are present in most cultures, some cultures hold more profound beliefs in superstition, fate, luck, and chance, which are presumably derived from cultural customs such as religion (Dickins and Thomas, 2016). Among other things, these beliefs are thought to extend to a given culture’s gambling practices (Raylu and Oei, 2004b; Papineau, 2005).

Members of the Chinese culture have shown profound beliefs in superstition, fate, luck, and chance (Raylu and Oei, 2004b; Papineau, 2005). Lam (2007) performed a naturalistic study which involved observing the gambling behaviors of Chinese baccarat players at a casino in Macau. He reported witnessing high levels of the illusion of control, inferred when players were observed shouting out specific words or phrases in hopes of influencing the chance of obtaining a smaller or larger numbered card. Ohtsuka and Chan (2010) examined the superstitious beliefs of Chinese problem and non-problem mahjong gamblers using self-report questionnaires. Although both gambler types reported superstitious beliefs, problem gamblers endorsed significantly more cognitive distortions regarding mahjong than non-problem gamblers. Compared to cultures in which beliefs in luck and chance are not as profound, members of the Chinese culture have shown greater beliefs specifically in the illusion of control compared to those of Caucasian decent (Oei et al., 2008). However, this is not always the case, and while superstitious beliefs can differ between cultures, certain beliefs may also be shared. For example, American and Chinese gamblers have been found to hold similarly strong superstitious beliefs regarding gambling rituals compared to Japanese and Korean gamblers, who do not tend to endorse this type of superstitious thinking (Kim et al., 2016).

Additional evidence of cultural differences in disordered gambling comes from differing prevalence rates of problem gambling between cultures. Differences in prevalence rates of disordered gambling are observed for many cultures, but also among those where beliefs in superstition, fate, luck, and chance are not as insidious and profound. In Netherlands, although 87% of individuals have reported gambling in their lifetime, only a small proportion exhibit gambling-related problems (De Bruin et al., 2006, as cited in Goudriaan et al., 2009). Approximately 1.0% of individuals 16 years of age and older are considered to have a lifetime gambling disorder in Netherlands; yearly prevalence rates of problem gambling are approximately 0.5%. This is similar to past year disordered gambling rates in nearby European countries such as Denmark (0.5%) yet comparatively lower than past year disordered gambling rates in Canada (1.2–2.2%) and the United States (1.7–4.6%; Williams et al., 2012).

While cultural customs and traditions may influence gambling practices, additional variables to be considered are the structural barriers imposed by laws which limit accessibility to gambling activities (Jacques et al., 2000; Raylu and Oei, 2004b). Different gambling laws and regulations within a particular culture may impact an individual’s values and beliefs around gambling (Raylu and Oei, 2004b). Certain gambling-related policies in Netherlands may contribute to the relatively lower disordered gambling rate compared to other countries such as Canada and the United States (De Bruin et al., 2001, as cited in Goudriaan et al., 2009). Since the year 2000 when stricter gambling laws were imposed, the number of slot machines in Netherlands has decreased. A primary goal from these more restrictive laws was to reduce “automatic,” persistent gambling behavior (De Bruin et al., 2001, as cited in Goudriaan et al., 2009, p. 191). As a part of these regulations, slot machines not currently in use were no longer permitted to display flashing lights or sounds, limiting their appeal to the potential user. Slot machines were no longer permitted in certain types of entertainment establishments such as bowling alleys and sports clubs, but continued to be allowed in pubs and restaurants, with these latter establishments limited to two slot machines each. Employees at gambling establishments received education on disordered gambling, including how to approach those who appeared to have a gambling problem or displayed problematic gambling behavior. Moreover, gamblers could voluntarily prohibit their own entry into certain gambling establishments (De Bruin et al., 2001, as cited in Goudriaan et al., 2009). However, the strict policies in 2000 do not necessarily apply to online gambling, which has become increasingly popular, forcing the privatization of the gambling industry and the liberalization of the gambling market since 2002 (Kingma, 2008).

Thus, while many individuals in Netherlands engage in gambling, a relatively lower proportion develop gambling problems relative to those in many other parts of Europe and in North America (De Bruin et al., 2006, as cited in Goudriaan et al., 2009). It is curious whether established risk factors such as cognitive distortions operate similarly in Dutch culture as in North America, where rates of problem gambling are relatively higher. However, it is difficult to assess whether certain gambling policies, such as those in Netherlands, create cross-cultural differences in gambling-related cognitive distortions. This is because many instruments assessing gambling-related cognitions are developed with, and validated on, North American gamblers. This calls into question the ability to generalize findings with these existing cognitive distortion measures to other cultures where values and beliefs may differ from those of mainstream North America.

### The Present Study

While the GCI has been validated in a large sample of Canadian gamblers, it has yet to be validated as a measure of distortions in other cultures. Further, while the relationship of the GCI subscales to gambling *problems* has been demonstrated (McInnes et al., 2014), the relation of GCI distortions to gambling *behaviors* has yet to be established. Additionally, the predictive validity of the GCI has yet to be determined. Thus, the purposes of the present study were to examine the concurrent and prospective relationships of the GCI scales to gambling *behaviors* and *problems* at baseline and over 1-month and 6-month follow-ups in a sample of Dutch gamblers. It was hypothesized that both Luck/Chance and Skill/Attitude distortions would be concurrently positively associated with both gambling *behaviors* and gambling-related *problems*. Moreover, it was hypothesized that baseline values on these two GCI subscales would be positively associated with gambling-related *behaviors* and *problems* 1-month and 6-months later. Findings different than those observed in North American gamblers (McInnes et al., 2014) in a sample of Dutch gamblers could broaden our understanding of the impact of cultural beliefs on gambling *behavior* and *problems* and elucidate whether established risk factors and correlates, in this case cognitive distortions, operate differently across cultures.

## Materials and Methods

### Participants

Participants were community-recruited through online gambling forums^1^^,^^2^, social media sites (i.e., Facebook and Twitter) and advertisements placed throughout the city of Amsterdam. To be eligible, participants were required to be 18 years of age or older, not attempting to abstain from gambling, and have gambled online or at a casino at least three times in the past 2 months (not including lottery tickets). Moreover, respondents were required to complete the study from distinct IP addresses to be eligible. This was to help eliminate any opportunities for fraud (i.e., a single participant completing the study more than once). Fifty-three participants were originally recruited. Four participants were excluded, one due to unfulfilled inclusion criteria and three due to repeat IP address issues and concerns about potential fraudulent data. The final sample was composed of 49 participants (all male) at baseline, 46 at the 1-month follow-up, and 41 at the 6-month follow-up (see **Table 1** for distribution of gambling risk). Our sample was primarily composed of low to moderate risk gamblers (see **Table 1**). At baseline, one participant completed only week one of the Gambling Timeline Followback (G-TLFB; Sobell and Sobell, 1992; Weinstock et al., 2004) and thus, this participant’s data was considered incomplete at this timepoint. Additionally, one participant solely entered data for the days in which they gambled at baseline but no other variable. Thus, for the G-TLFB at baseline, 48 participants have complete data for days gambled and 47 have complete data for time spent gambling. At the 1-month follow-up, two participants failed to complete the GCI and thus for this measure, there were 44 participants. At baseline, participants ranged in age from 20 to 59 years old (*M* = 30.8, *SD* = 9.0). Once eligible, participants completed Dutch translations of the following questionnaires: the Problem Gambling Severity Index (PGSI) of the Canadian Problem Gambling Index (CPGI; Ferris and Wynne, 2001; McCready and Adlaf, 2006) which assessed level of gambling problems, the GCI (Holub, 2003; McInnes et al., 2014) to assess gambling-related cognitive distortions, the 30-day G-TLFB (Sobell and Sobell, 1992; Weinstock et al., 2004) to assess gambling behavior, and the first question of the South Oaks Gambling Screen (SOGS; Lesieur and Blume, 1987) to describe the types of gambling games played by participants.

**Table 1:** Distribution of gambling risk across baseline (T1), 1-month (T2), and 6-months (T3).

	Non-problem gambler		Low levels of problems		Moderate levels of problems		Problem gambler	
	*n*	%	*n*	%	*n*	%	*n*	%
T1	8	16.3	18	36.7	18	36.7	5	10.2
T2	9	19.6	14	30.4	21	45.7	2	4.3
T3	9	22.0	16	39.0	13	31.7	3	7.3

### Measures

#### Translation of Measures

Dutch translations of the SOGS and PGSI were used in the present study. The GCI was translated from English to Dutch and then back-translated from Dutch to English by two independent research associates. The first individual was a native Dutch speaker who was fluent in English and familiar with the contents of the GCI and the second individual was a native (American) English speaker who was also fluent in Dutch. The comparison of the back-translated version of the GCI to the original English version was acceptable and required only minor revisions in phrasing. A research associate also translated the G-TLFB from English to Dutch. The G-TLFB did not require any back-translation procedure as it is very simple and explicitly asks specific questions.

#### Severity of Gambling-Related Problems

The PGSI of the CPGI was administered to determine participant’s severity of gambling problems. This measure is composed of nine items (e.g., “How often have you bet more than you could really afford to lose?”; “How often has your gambling caused you any health problems, including stress or anxiety?”). Each question is rated on a 4-point Likert scale with responses being 0 (*never*), 1 (*sometimes*), 2 (*most of the time*), and 3 (*almost always*). All nine items are summed with higher scores indicating greater gambling-related problems (Currie et al., 2013). This summed score creates four categories of gambling-related risk: non-problem gambling (score of 0), low levels of gambling problems (score of 1 or 2), moderate levels of gambling problems (score of 3–7), and problem gambling (score of 8 or more; Ferris and Wynne, 2001). The PGSI has demonstrated good internal consistency (α = 0.84; Ferris and Wynne, 2001) and test–retest reliability over a 3- to 4-week period (*r* = 0.78). It has also shown good convergent validity with the SOGS and *DSM-IV* criteria for pathological gambling (American Psychiatric Association, 1994; Ferris and Wynne, 2001). In the present study, the PGSI demonstrated acceptable internal consistency at baseline (α = 0.74) and 6-months (α = 0.74), and marginally acceptable internal consistency at 1-month (α = 0.66). Moreover, it demonstrated good test–retest reliability over 1-month [*r*(46) = 0.73, *p* < 0.001] and 6-months [*r*(41) *=* 0.65; *p* < 0.001] in the present study.

#### Gambling-Related Cognitive Distortions

The original GCI is a 40-item measure that assesses gambling-related cognitive distortions regarding Luck/Chance and Skill/Attitude (Holub, 2003; McInnes et al., 2014). Each item is rated on a 4-point Likert scale ranging from 1 (*strongly agree*) to 4 (*strongly disagree*). Items were reverse scored so that higher scores indicate higher levels of given cognitive distortions. In the present study, we administered the 40-item version but scored the measure based on the refined 33-item version which is embedded within the original (McInnes et al., 2014). The refined version has 14 items for the Luck/Chance subscale and 19 items for the Skill/Attitude subscale. An example of a question related to Luck/Chance cognitive distortions is: “I can tell when I am lucky or am having a lucky day, and that is a good day to gamble.” An example of a question assessing cognitive distortions related to Skill/Attitude is: “I can analyze my wins to give me strategies to make me a better gambler.” To score the subscales of the 33-item GCI, items are first converted from a 1–4 to a 0–3 scale; then items pertaining to each subscale are summed and divided by the maximal possible summed score to obtain a score between 0 and 1. A score closer to 1 indicates greater cognitive distortions while a score closer to 0 indicates less cognitive distortions. To score the Luck/Chance subscale, each of the relevant 14 items were summed; this sum was then divided by 42 (the maximum possible Luck/Chance sum). To score the Skill/Attitude subscale, each of the 19 items was summed; this sum was then divided by 57 (the maximum possible Skill/Attitude sum). The Luck/Chance and Skill/Attitude subscales have shown marginally acceptable to good internal reliability (α = 0.67–0.87) and good concurrent validity with the SOGS (*r*’s = 0.41–0.43) and PGSI in Canadian samples (*r*’s = 0.25–0.36; significant at *p* < 0.05; McInnes et al., 2014).

#### Gambling Behavior

The G-TLFB is a 30-day retrospective calendar that asks participants to reflect upon and report various gambling-related behaviors they have engaged in during the past month. The present study looked specifically at the following behaviors: number of days gambled and time spent gambling. Initially, money risked gambling was included as an outcome measure. However, this measure was poorly correlated, and often not correlated, with itself and the other G-TLFB items and thus was not included in the present study. Anecdotally, participants find money risked a hard concept to understand and the person administering the measure often needs to explain, with examples, what is meant by money risked. It is possible that in the online format, participants interpreted this item differently and this added measurement error and obscured any expected relations.

Participants were instructed to enter data on their gambling behavior for each day that they gambled. The G-TLFB exhibits good test–retest reliability over a 2-week period (*r*’s = 0.74–0.96) and good concurrent validity with the SOGS (*r*’s = 0.30–0.32; Lesieur and Blume, 1987; Weinstock et al., 2004). In the present study, days spent gambling showed good test–retest reliability over 1-month [*r*(46) = 0.57; *p* < 0.001] and 6-months [*r*(41) = 0.43; *p* = 0.003]. As well, time spent gambling showed good test–retest reliability over 1-month [*r*(45) = 0.72; *p* < 0.001] and 6-months [*r*(41) = 0.36; *p* = 0.010].

#### Frequency of Games Played

The first question of the SOGS was used for sample description purposes to assess the frequency with which participants engaged in certain types of gambling (Lesieur and Blume, 1987). Participants were asked to indicate the degree to which they have partaken in particular forms of gambling in their lifetime, with responses being “*not at all,”* “*less than once a week,”* and “*once a week or more.”*

#### Statistical Analysis

Pearson product moment correlations and Spearman’s Rank correlations were performed in IBM SPSS Statistics (version 22) between the GCI subscales (i.e., Luck/Chance and Skill/Attitude) and gambling problem and behavior indices from the PGSI and G-TLFB, respectively. Pearson’s correlations were followed with a test of differences in dependent correlations using Steiger’s *Z*-test of parametric correlations. One-tailed tests were used, as directional predictions had been made *a priori*. Internal consistency estimates for each GCI subscale were also performed in SPSS by calculating Cronbach’s alpha. Cohen’s (1992) conventions were used to judge the magnitude of effect sizes for the correlations and the differences between correlations in the present study. Cohen’s conventions are as follows: small (0.10 < *r* < 0.30); medium (0.30 < *r* < 0.50); large (*r* < 0.50).

### Procedure

The present study was carried out in accordance with the recommendations of the University of Amsterdam and the Dalhousie University Research Ethics Boards with informed consent from all subjects. The protocol was approved by the University of Amsterdam and the Dalhousie University Research Ethics Boards. The participants completed the screening, informed consent, questionnaires, and debriefing online in their homes. Interested participants clicked on the link to the study website displayed on banners and advertisements. This link directed the participant to the study website where they created an account and completed eligibility screening. Once deemed eligible, participants were directed to a page detailing informed consent. As the study was performed online, informed consent involved checking boxes for statements that detailed the informed consent. Checking these boxes indicated that the participant understood each statement and that they made an informed decision to participate. All participants gave informed consent in accordance with the Declaration of Helsinki. Once the participant consented, they were invited to begin the study. The following questionnaires were administered at baseline: a demographics questionnaire, the PGSI of the CPGI, the GCI, the G-TLFB, and the first question of the SOGS for descriptive purposes. The following questionnaires were administered at the 1-month and 6-month follow-up: the PGSI of the CPGI, the GCI, and the G-TLFB. Each session (baseline, 1-month follow-up, and 6-month follow-up) lasted approximately 25 min and following its completion, the participant received a claim code for a €17.80 (∼$25.00 Canadian) Bol.com vouchers as remuneration. All the questionnaires were completed online using the platform Qualtrics (Qualtrics, 2016).

## Results

### Descriptive Statistics

Means, standard deviations, and ranges for measures of gambling problems, gambling behaviors (i.e., days spent gambling and time spent gambling in minutes), and gambling-related cognitive distortions (i.e., Luck/Chance and Skill/Attitude distortions) are available in **Table 2**. Values provided for the GCI subscales are based on the 0–1 rescaling of the measure.

**Table 2:** Means, standard deviations, and ranges of Luck/Chance and Skill/Attitude Gambling Cognitions Inventory (GCI) subscales and outcome measures of gambling severity (PGSI) and behavior (G-TLFB) at baseline (T1), 1-month (T2), and 6-months (T3).

	T1				T2			T3		
	*M*	*SD*	Range		*M*	*SD*	Range		*M*	*SD*	Range	
Variable			Possible	Actual			Actual			Actual
PGSI	3.12	2.83	0 – 27	0 – 11	2.89	2.46	0 – 10	2.76	2.82	0 – 12
**G-TLFB**								
Days	9.00	6.46	0 – 31	0 – 31	9.61	8.91	0 – 31	6.66	6.91	0 – 31
Time (min)	1,260.17	1,211.86	–	0 – 5,768	1,425.13	1,802.21	0 – 9,563	1,051.44	1,271.32	0 – 6,195
**GCI**								
Luck/Chance	0.15	0.15	0 – 1	0 – 0.57	0.23	0.21	0 – 0.71	0.21	0.20	0 – 0.81
Skill/Attitude	0.44	0.20	0 – 1	0 – 0.75	0.50	0.18	0 – 0.77	0.47	0.23	0 – 0.96

In the present study, frequent playing of skills games (e.g., cards) was much more common than frequent playing of chance games (e.g., 98% played cards versus 14% played slot machines once a week or more), as indexed by the first item of the SOGS.

### Psychometric Properties of the Gambling Cognitions Inventory

The Skill/Attitude and Luck/Chance subscale were moderately inter-correlated at baseline [*r*(49) = 0.56, *p* < 0.001] and 6-months [*r*(41) = 0.57, *p* < 0.001] and strongly inter-correlated at 1-month [*r*(44) = 0.67, *p* < 0.001]. The Skill/Attitude and Luck/Chance subscales also demonstrated good to excellent internal consistency at baseline (α’s = 0.90 and 0.87, respectively), 1-month (α’s = 0.89 and 0.93, respectively), and 6-months (α’s = 0.94 and 0.92, respectively). Lastly, the test–retest reliability of the Luck/Chance subscale [*r*(44) = 0.79, *p* < 0.001] and Skill/Attitude subscale [*r*(44) = 0.75, *p* = 0.001] from baseline to 1-month follow-up were both significant, demonstrating strong stability over 1-month. At baseline to 6-months, the test–retest reliability of the Luck/Chance subscale [*r*(41) = 0.73, *p* < 0.001] and Skill/Attitude subscale [*r*(41) = 0.77, *p* < 0.001] were significant, demonstrating strong stability over 6-months.

### Gambling Problems

Luck/Chance distortions were positively related to gambling problems at all timepoints (baseline, 1-month, and 6-months; see **Table 3**, left column) and these relations were medium (baseline and 6-months) and large (1-month) in magnitude.^3^ Cognitive distortions regarding Skill/Attitude were unrelated to gambling problems at all three timepoints.

**Table 3:** Pearson Product Moment Correlations between the Luck/Chance and Skill/Attitude Gambling Cognitions Inventory (GCI) Subscales at baseline (T1) with outcome measures of gambling severity (PGSI) and behavior (G-TLFB) at baseline (T1), 1-month (T2), and 6-months (T3).

	Outcome Variable		
T1 GCI Subscales	PGSI	G-TLFB	
Outcome wave		Days	Time
**Luck/Chance**			
T1 outcome	*r*(49) = 0.43, *p* = 0.001^∗∗^	*r*(48) = 0.10, *p* = 0.247	*r*(47) = 0.21, *p* = 0.076
T2 outcome	*r*(46) = 0.51, *p* < 0.001^∗∗∗^	*r*(46) = 0.08, *p* = 0.310	*r*(46) = 0.08, *p* = 0.308
T3 outcome	*r*(41) = 0.44, *p* = 0.002^∗∗^	*r*(41) = 0.31, *p* = 0.025^∗^	*r*(41) = 0.18, *p* = 0.126
**Skill/Attitude**			
T1 outcome	*r*(49) = 0.03, *p* = 0.426	*r*(48) = 0.29, *p* = 0.023^∗^	*r*(47) = 0.36, *p* = 0.007^∗^
T2 outcome	*r*(46) = 0.06, *p* = 0.338	*r*(46) = 0.24, *p* = 0.053^†^	*r*(46) = 0.18, *p* = 0.118
T3 outcome	*r*(41) = 0.12, *p* = 0.229	*r*(41) = 0.34, *p* = 0.014^∗^	*r*(41) = 0.25, *p* = 0.062^†^

*^∗∗∗^*p* < 0.001, ^∗∗^*p* < 0.01, ^∗^*p* < 0.05. ^†^Marginally significant. All one-tailed tests.*

Steiger’s *Z*-tests showed the correlation between Luck/Chance distortions and gambling problems was stronger than the correlation between Skill/Attitude distortions and gambling problems at baseline (*z* = 3.02, *p* = 0.002), 1-month (*z* = 3.33, *p* < 0.001), and 6-months (*z* = 2.22, *p* = 0.026). All effect sizes of these correlational differences were medium in magnitude.

### Gambling Behavior

#### Days Spent Gambling

Baseline endorsement of Luck/Chance distortions were not concurrently related to days spent gambling at baseline nor were they prospectively associated with days spent gambling at 1-month (see **Table 3**, middle column). However, baseline Luck/Chance scores were positively related to days spent gambling at 6-months, and this effect size was moderate in magnitude. Skill/Attitude distortions were positively related to days spent gambling at all timepoints, being significantly related at baseline and 6-months and marginally related at 1-month. The effect sizes of these correlations were small at both baseline and 1-month but moderate at 6-months.

Steiger’s *Z*-tests showed that the correlation between baseline Skill/Attitude distortions and days spent gambling was no stronger than the correlation between baseline Skill/Attitude distortions and gambling problems at baseline (*z* = -1.30, *p* = 0.195), 1-month (*z* = *-*0.81, *p* = 0.419), or 6-months (*z* = *-*1.19, *p* = 0.236). Moreover, the correlation between baseline Skill/Attitude distortions and days spent gambling was no stronger than the correlation between baseline Luck/Chance distortions and days spent gambling at baseline (*z* = -1.40, *p* = 0.16), 1-month (*z* = -1.18, *p* = 0.24), or 6-months (*z* = -0.25, *p* = 0.81). However, the correlation between baseline Luck/Chance and 1-month gambling problems was stronger than the correlation of baseline Luck/Chance with 1-month days spent gambling (*z* = 2.10, *p* = 0.035; compare **Table 3**, left and middle columns) and the effect size of this correlational difference was medium in magnitude.

Because baseline Luck/Chance distortions were related to both gambling problems and gambling behavior (days spent gambling) at 6-months, we also conducted *post hoc* partial correlations to examine possible unique relations of this form of distortion with gambling problems. *Post hoc* partial correlations revealed, when controlling for days spent gambling at 6-months, baseline Luck/Chance distortions continued to be positively related to 6-month PGSI scores (*r_ab.c_* = 0.39, *p* = 0.006). However, the relationship between baseline Luck/Chance distortions and days spent gambling at 6-months was no longer significant when PGSI scores at 6-months were controlled in a *post hoc* partial correlation (*r_ab.c_* = 0.22, *p* = 0.084).

#### Time Spent Gambling

Baseline Luck/Chance distortions were not related to time spent gambling at any timepoint (see **Table 3**, right column). Baseline Skill/Attitude distortions were concurrently, positively, related to time spent gambling at baseline and marginally, positively, related to time spent gambling at 6-months. These correlations were moderate and small in magnitude, respectively. Skill/Attitude distortions were not related to time spent gambling at 1-month.

Steiger’s *Z*-tests showed the correlation between baseline Skill/Attitude distortions and time spent gambling was no stronger than the correlation between baseline Skill/Attitude distortions and gambling problems at baseline (*z* = *-*1.67, *p* = 0.095), 1-month (*z* = -0.54, *p* = 0.593), or 6-months (*z* = *-*0.64, *p* = 0.521). Moreover, the correlation between baseline Skill/Attitude distortions and time spent gambling was no stronger than the correlation between baseline Luck/Chance distortions and time spent gambling at baseline (*z* = -1.08, *p* = 0.28), 1-month (*z* = -0.72, *p* = 0.47), or 6-months (*z* = -0.42, *p* = 0.68). However, the correlation between baseline Luck/Chance and 1-month gambling problems was stronger than the correlation of baseline Luck/Chance with 1-month time spent gambling (*z* = 2.16, *p* = 0.03; compare **Table 3**, left and right columns) and the effect size of this difference was medium in magnitude.

### Summary

Luck/Chance distortions were more strongly related to problems than to behaviors, with the differences in strength being medium in magnitude. Luck/Chance was also more strongly related to problems than was Skill/Attitude, with the effect size of this difference being medium in magnitude. Although Skill/Attitude distortions were related to gambling behaviors but not to gambling problems, skill distortions were not more strongly related to behaviors than they were to problems. Further, there was no evidence to suggest that Skill/Attitude distortions were more strongly related to gambling behaviors than were Luck/Chance distortions.

## Discussion

The present study investigated the validity of the GCI as a measure of cognitive distortions in a sample of Dutch gamblers. Moreover, it investigated the concurrent and prospective relationship of the GCI to gambling *problems* and *behaviors* at baseline and over 1-month and 6-months. The findings provide initial support for the cross-cultural validity of the measure in tapping aspects of gambling-related cognitive distortions that show important relationships to gambling outcomes. In a sample of Dutch gamblers, as baseline Luck/Chance distortions increased, so did concurrent gambling-related *problems*. Having greater cognitive distortions related to one’s own luck were also associated with greater gambling-related problems 1- and 6-months later. These baseline Luck/Chance distortions were found to be uniquely associated with gambling-problems. While baseline luck distortions were also associated with a greater number of days spent gambling at the 6-month follow-up, this relation did not persist when 6-month gambling problems were controlled. In contrast, higher levels of baseline Skill/Attitude distortions were concurrently associated with a greater number of days spent gambling and increased time spent gambling. Believing one has greater gambling-related skill at baseline was significantly associated with greater days spent gambling at the 6-month follow up. Moreover, baseline skill beliefs were also marginally associated with days spent gambling at the 1-month follow-up, and with greater time spent gambling at the 6-month follow-up.

Our findings partially provide a cross-cultural replication of the GCI as being related to gambling problems in Dutch gamblers. Our observed relationship of Luck/Chance distortions to gambling problems is consistent with previous literature with Canadian gamblers, where increased Luck/Chance distortions were related to increased severity of gambling problems (McInnes et al., 2014). Similar to the present study, these effects were of moderate strength (McInnes et al., 2014). This finding also fits with the broader literature using other cognitive distortion measures that suggest a greater endorsement of gambling-related false beliefs is associated with a greater severity of gambling-related problems (e.g., Cunningham et al., 2014). Thus, we can conclude this aspect of McInnes et al.’s (2014) findings with Canadian gamblers appears to be generalizable to Dutch gamblers. However, we did not replicate McInnes et al.’s (2014) findings that Skill/Attitude distortions were concurrently associated with severity of gambling problems. In fact, in our Dutch sample, the relation between Luck/Chance distortions and gambling problems was significantly stronger than the relation between Skill/Attitude distortions and gambling problems – a difference of moderate magnitude. Moreover, the relationship between baseline Luck/Chance distortions and gambling problems at 6-months persisted even after controlling for days spent gambling at 6-months, suggesting these distortions are uniquely related to future gambling problems even after controlling for gambling behavior. These findings call for further research to determine what may underlie the relationship between gambling problems and distortions related to luck and how these distortions might put people at risk for gambling problems over-and-above putting them at risk for excessive gambling behaviors.

One possible reason why Luck/Chance distortions, but not Skill/Attitude distortions, were related to current and future gambling *problems* in the present study of Dutch gamblers pertains to the nature of our Dutch sample. While the entire sample of Canadian gamblers in McInnes et al. (2014) consisted of problem and disordered gamblers, the present Dutch study included non-problem as well as problem gamblers. Overall, the average severity of gambling problems in our sample was in the moderate risk range (mean PGSI score of approximately 3). It is possible that relations of Skill/Attitude distortions to gambling problems may only emerge at relatively higher levels of gambling problems, and perhaps the level of problems was not sufficiently high in our sample to reveal relations with Skill/Attitude distortions.

The present study extended the results found by McInnes et al. (2014) by examining the relations of GCI subscale scores to gambling *behaviors* – an outcome not previously examined in relation to GCI scores in any cultural group. The findings of the present study fit with the results of Joukhador et al. (2004) using an alternate measure of gambling-related cognitive distortions whereby among a sample of machine gamblers, those with greater gambling-related cognitive distortions spent more time gambling and participated in more gambling sessions each week compared to those with fewer cognitive distortions. While we found Skill/Attitude distortions were related to gambling behaviors and generally not to gambling problems, the correlation differences of Skill/Attitude distortions with gambling behaviors as the outcome were not significantly greater than the correlation of Skill/Attitude distortions with gambling problems. Nor were the correlations between Skill/Attitude distortions with gambling behaviors significantly greater than the correlation of Luck/Chance distortions with gambling behaviors. This may have been due to the small sample size and associated weak power to detect such correlational differences in the present study, as discussed further in the limitations section. While admittedly a less robust finding than the unique and moderate sized relationship of luck distortions to gambling problems, our correlational findings provide modest support for a relationship between skill distortions and increased gambling behaviors, a relationship not found for luck distortions. Baseline Luck/Chance distortions were unrelated to either gambling behavior at baseline or 1-month follow-up in our Dutch sample of gamblers. While baseline Luck/Chance distortions were related to days spent gambling at 6-months, *post hoc* analyses revealed this effect did not persist when gambling problems at 6-months were statistically controlled.

One possible reason why Skill/Attitude distortions, as opposed to Luck/Chance distortions, were predominantly related to gambling *behaviors* but not to gambling *problems* in our study pertains to the types of games favored by our sample of Dutch gamblers. It has been suggested that games of skill bring about persistent gambling *behavior* compared to games of luck (Dickerson, 1993). In fact, increasing an individual’s perceived skill over a game has been shown to result in greater gambling *behavior* (Langer, 1975). Myrseth et al. (2010) found that gamblers who solely preferred games of skill evidenced greater illusion of control (similar to Skill/Attitude cognitive distortions) compared to those who solely preferred games of chance. Perhaps those who engage in more games of skill believe they have greater control over the game and thus perceive they have greater control over their gambling, resulting in greater gambling *behavior*. The Dutch gamblers in the present study were community-recruited through online websites, posters, and gambling forums. Particularly on gambling forums, gamblers discuss certain strategies or perceived skill related to gambling which may have caused a selection bias, resulting in the recruitment of those who predominantly played skill-based games. In support of this possibility, frequent playing of skill games (e.g., playing cards) once a week or more was much more common than frequent playing of chance games (e.g., playing slots) among the present study’s gamblers (98% vs 14%, respectively). However, it is important to emphasize that this explanation is speculative.

While it is plausible certain methodological and sampling differences may have brought about the differences between our study and the findings of McInnes et al. (2014), the divergences may be the result of a true cross-cultural difference in cognitive distortions and/or gambling practices. Unlike McInnes et al. (2014) Canadian study, we did not observe a relationship between Skill/Attitude distortions and *problems* in our Dutch sample. This does not indicate the GCI Skill/Attitude scale is an invalid measure of cognitive distortions in Dutch gamblers, as Skill/Attitude belief scores were related to gambling *behaviors*. Rather, it is possible that differences in gambling practices and policies ascribed by different cultures resulted in inherently different gamblers in Netherlands than those in Canada (Raylu and Oei, 2004b; Papineau, 2005; Oei et al., 2008). Perhaps distorted beliefs about skill are more important for the development and maintenance of gambling-related problems in Canadian as opposed to Dutch gamblers due to certain cultural or structural differences. For example, according to Hofstede’s (2001) cultural ratings (see also Hofstede et al., 2010), Canada is a substantially more masculine culture (i.e., emphasis on ambition and accumulation of wealth) than Netherlands (i.e., relative masculinity scores of 52 vs. 14). It is possible that an individual with high levels of skill distortions might be more likely to develop problems with gambling in a culture that places relatively more emphasis on material wealth and winning (i.e., in a more ‘masculine’ culture).

### Limitations

Unexpectedly, only male gamblers were recruited and thus, we are unable to generalize our findings to Dutch female gamblers. While gambling is more common in men than women (Welte et al., 2002), we were neither expecting nor intending to recruit only men into the present study. Previous empirical literature has found numerous differences in gambling practices between males and females. For example, males tend to prefer games requiring skill (e.g., sports betting or cards) while females tend to prefer playing games of luck or chance where no such skill is involved (e.g., slot machines; Toneatto et al., 1997). Indeed, skill-based games were the games predominantly played by participants in our study. Thus, we may have had insufficient variability in Skill/Attitude distortions to see any relation with gambling-related problems. However, this seems unlikely given there was sufficient variability in Skill/Attitude distortions to see relations with gambling behavior. Future research should be sure to recruit an equal distribution of male and female gamblers to allow for between-gender comparisons on different types of gambling-related cognitive distortions.

As alluded to earlier, a possible limitation concerns the relatively small sample size and the consequent impact on power. A *post hoc* power analysis showed that to obtain power of 0.8 for a moderate effect size of *r* = 0.30, 67 participants would have been needed. Thus, with a sample size of 49 participants, the present study was adequately powered to detect large effects but underpowered to detect small effects. Attrition and unmet inclusion criteria further contributed to power issues for detecting statistically significant relations of skill distortions and gambling behaviors at the follow-ups, or for demonstrating significantly stronger correlations of skill distortions with gambling behavior relative to other correlations. Nonetheless, we did detect one significant effect and two marginal trends that are suggestive of the utility of Skill/Attitude distortions in understanding prospective gambling behaviors. Moreover, due to the relatively small sample size in the present study, we were unable to perform Exploratory or Confirmatory Factor Analyses on the GCI and thus unable to fully demonstrate cross-cultural validity of this scale. However, the Cronbach’s alphas for the GCI in the present study indicate good internal consistency of the subscales based on the factorial structure of the GCI reported by McInnes et al. (2014). While the two-factor structure of the GCI has been previously established by McInnes et al. (2014), the validity of this distinct two-factor structure in other cultures has yet to be been demonstrated. Thus, a larger study is necessary to confirm the cross-cultural validity of the GCI’s factor structure. Further, a larger sample size would permit the use of multiple regression analyses controlling for baseline levels of the outcomes to establish whether GCI cognitive distortions predict changes in gambling outcomes over time. While our study suggests greater baseline cognitive distortions are predictive of greater gambling problems and behaviors over time, it could be that gambling behaviors are predictive of escalations in cognitive distortions over time as opposed to the reverse. However, temporal directionality consistent with cognitive theory (Ladouceur and Walker, 1996) has been previously established in a longitudinal study with a sample of 1,000 Canadian gamblers. Specifically, Yakovenko et al. (2016) found changes in cognitive distortions reliably preceded and predicted increases in gambling behavior, and that this path was stronger than the converse path from gambling behavior to increased cognitive distortions over time. Future longitudinal research should examine such temporality in the Dutch population and with the GCI to determine if Yakovenko et al.’s (2016) directional results are true cross-culturally and for the distortions measured by the GCI.

As the present study was performed online in the comfort of the participants’ homes rather than in a controlled, laboratory setting, we are unable to ensure participants completed each component of the study as directed (e.g., alone without distraction). However, the research team did not receive any inquiries for clarification from any of the participants and at least some of the McInnes et al. (2014) results were replicated in the present sample, attesting to their validity.

Lastly, due to the differences between our sample and the one studied in McInnes et al. (2014), the potential cross-cultural differences highlighted in this paper should be interpreted with caution. Future studies directly comparing gambler samples with similar characteristics in Canada, Netherlands, and other countries, will yield results that can be interpreted as cross-cultural differences with higher confidence.

## Conclusion

Cognitive distortions have been known to play an important role in creating and maintaining disordered gambling (Ladouceur and Walker, 1996). In fact, they are such a fundamental contributor to disordered gambling that they are often targeted in gambling treatment (Ladouceur et al., 1998; Walker et al., 2006; Fortune and Goodie, 2012). Targeting these cognitive distortions in treatment has shown positive results in reducing gambling behaviors and problems (Walker et al., 2006; Fortune and Goodie, 2012). The results of our study suggest there are distinct cognitive correlates that may be associated with certain aspects of disordered gambling, at least in Dutch gamblers. Dutch gamblers who endorse greater Luck/Chance distortions may not gamble more frequently or spend greater amounts of time gambling overall, yet when they do gamble they may engage in more risky gambling as a function of their belief in luck. Dutch gamblers who believe they have greater gambling skill may wager more frequently and spend more time gambling yet this may not necessarily be associated with developing gambling-related problems. Beyond excessive gambling involvement, how and why an individual engages in gambling activities may be an important contributor to developing and/or maintaining excessive or problem gambling (Stewart and Zack, 2008). Moreover, cultural differences may further contribute to disordered gambling by influencing the types of erroneous beliefs gamblers hold. While beliefs in luck and skill both contribute to gambling problems in Canadian gamblers (McInnes et al., 2014), Dutch gamblers do not show the same relation of skill distortions to gambling problems. In Dutch gamblers, beliefs about skill may be more important for contributing to excessive gambling behaviors while beliefs about luck may have a greater influence on developing gambling problems. Importantly, in conceptualizing and understanding cognitive distortions as being composed of two distinct yet overlapping dimensions (i.e., Luck/Chance and Skill/Attitude) each of which have distinct gambling outcome correlates (i.e., gambling problems and behaviors, respectively), we might further our understanding of the cognitive underpinnings of excessive and problem gambling.

## Author Contributions

All authors listed have made a substantial, direct and intellectual contribution to the work, and approved it for publication.

## Conflict of Interest Statement

Research funded through the National Center for Responsible Gaming is provided by an array of different sources, one of which is the gambling industry. Thus, while the gambling industry has indirectly funded this research through contributions to our funder, the NCRG, they had no role in the conduct of this study, the interpretation of findings, or the decision to publish.

## Footnotes

1 http://Voetbalweddenschappen.com

2 http://onlinegokforum.nl

3 Given violations of assumptions of normality, Spearman’s rank correlations were also performed for each variable at all timepoints. The pattern of findings were similar to those reported using Pearson correlations where Luck/Chance distortions were related to problems both concurrently and prospectively while distortions related to Skill/Attitude were related to behaviors both concurrently and prospectively, yet not problems.
